# Indian *Bt* Cotton Varieties Do Not Affect the Performance of Cotton Aphids

**DOI:** 10.1371/journal.pone.0004804

**Published:** 2009-03-12

**Authors:** Nora C. Lawo, Felix L. Wäckers, Jörg Romeis

**Affiliations:** 1 Agroscope Reckenholz-Tänikon Research Station ART Zurich, Zurich, Switzerland; 2 Lancaster University, Lancaster, United Kingdom; Cairo University, Egypt

## Abstract

Cotton varieties expressing Cry proteins derived from the soil bacterium *Bacillus thuringiensis* (*Bt*) are grown worldwide for the management of pest Lepidoptera. To prevent non-target pest outbreaks and to retain the biological control function provided by predators and parasitoids, the potential risk that *Bt* crops may pose to non-target arthropods is addressed prior to their commercialization. Aphids play an important role in agricultural systems since they serve as prey or host to a number of predators and parasitoids and their honeydew is an important energy source for several arthropods. To explore possible indirect effects of *Bt* crops we here examined the impact of *Bt* cotton on aphids and their honeydew.

In climate chambers we assessed the performance of cotton aphids, *Aphis gossypii* Glover (Hemiptera: Aphididae) when grown on three Indian *Bt* (Cry1Ac) cotton varieties (MECH 12, MECH 162, MECH 184) and their non-transformed near isolines. Furthermore, we examined whether aphids pick up the *Bt* protein and analyzed the sugar composition of aphid honeydew to evaluate its suitability for honeydew-feeders.

Plant transformation did not have any influence on aphid performance. However, some variation was observed among the three cotton varieties which might partly be explained by the variation in trichome density. None of the aphid samples contained *Bt* protein. As a consequence, natural enemies that feed on aphids are not exposed to the Cry protein. A significant difference in the sugar composition of aphid honeydew was detected among cotton varieties as well as between transformed and non-transformed plants. However, it is questionable if this variation is of ecological relevance, especially as honeydew is not the only sugar source parasitoids feed on in cotton fields.

Our study allows the conclusion that *Bt* cotton poses a negligible risk for aphid antagonists and that aphids should remain under natural control in *Bt* cotton fields.

## Introduction

Heliothine caterpillars, such as *Helicoverpa* spp. (Lepidoptera: Noctuidae) or *Heliothis virescens* (Fabricius) (Lepidoptera: Noctuidae), and the pink bollworm, *Pectinophora gossypiella* (Saunders) (Lepidoptera: Gelechiidae), are key pests of cotton worldwide. To control these polyphagous herbivores, farmers routinely use large amounts of broad-spectrum chemical insecticides, killing many non-target arthropods in the process. However, since heliothine caterpillars have a history of developing resistance to almost all the insecticides used for their control [1]–[3], alternative control methods have to be developed. One option is the use of insect-resistant genetically engineered (GE) varieties expressing lepidopteran-active Cry proteins derived from the soil bacterium *Bacillus thuringiensis* (*Bt*). So-called *Bt* cotton plants are grown commercially since 1996. Most of today's varieties express the *Bt* protein Cry1Ac either alone or in combination with Cry2Ab, protecting plants from damage by the main pest Lepidoptera [4].

In 2007, *Bt* cotton was grown in nine countries (USA, Argentina, Brazil, India, China, South Africa, Australia, Mexico, and Colombia) [5]. As cotton is among the most intensively sprayed of all field crops, the introduction of *Bt* cotton has had a tremendous impact in terms of reducing insecticide use resulting in economic, environmental and human health benefits [6], [7]. The first eleven years of *Bt* cotton production (1996–2006) have resulted in a 22.9% reduction in insecticide active ingredient application in cotton world wide [8]. Insecticide reductions were most significant in India and China where the improved pest control also related to significant increases in yield [6], [7], [9]. In India, *Bt* cotton hybrids expressing the *cry1Ac* gene are cultivated on an increasing area since their introduction in 2002. Almost tripling the area to 3.8 million hectares in 2006, India became the largest *Bt* cotton growing country in the world and in 2007, 131 *Bt* cotton hybrids were grown on a total of 6.2 million hectares [5].

Due to the reduction of broad spectrum insecticides in *Bt* cotton, herbivores which are not targeted by the *Bt* protein survive and occasionally reach pest status [4]. To retain the biological control function provided by naturally occurring antagonists of herbivores –i.e. predators and parasitoids– and to prevent non-target pest outbreaks, the potential risk that GE crops may pose to natural enemies is addressed as part of the environmental risk assessment prior to the commercial release of any novel GE crop [10], [11]. Several studies examined the effect of *Bt* crops on herbivores and arthropod natural enemies in recent years confirming the highly selective mode of action of the deployed *Bt* Cry proteins [12], [13].

Aphids generally play an important role in agricultural food webs since they serve as hosts or prey for a variety of parasitoids and predators. Consequently, the question whether aphids are affected by the *Bt* crop and whether they expose their natural enemies to the plant-expressed *Bt* protein is of high relevance. Studies available to date provide no evidence that *Bt* crops, expressing Cry1A proteins for the control of pest Lepidoptera, cause direct adverse effects on aphids [14], [15]. This is not surprising, since the *Bt* protein does not appear to be ingested by aphids which feed on phloem-sap [15]–[20]. Surprisingly, two studies have reported considerable amounts of *Bt* Cry proteins in aphid samples [21], [22].

Further, aphids and other phloem feeders produce honeydew which is an important source of carbohydrates for sugar feeding arthropods, including hymenopteran parasitoids and aphid predators [23], [24]. Sugars can enhance parasitoid reproductive fitness by increasing their longevity, fecundity and/or parasitism rate [25]–[30]. However, honeydew can be a relatively unsuitable sugar source as a result of unfavorable sugar composition [27], [29], [30]. Honeydew nutrient composition could also be altered as a result of plant transformation.

Therefore, we investigated in standardized laboratory bioassays if the performance of the cotton aphid, *Aphis gossypii* Glover (Hemiptera: Aphididae), was affected on three Indian *Bt* cotton varieties (MECH 12, MECH 162, MECH 184), expressing the Cry1Ac protein and their corresponding non-transformed near isolines. We clarified whether aphids pick up the *Bt* protein and we examined several aphid life-table parameters,. Furthermore, to gain insight into the impact of *Bt* on the nutrient composition of aphid honeydew the sugar composition of honeydew was examined.

## Results

### Aphid performance

With one exception, statistical analyses showed neither a *Bt*-transformation nor a cotton variety effect for any of the aphid life-table parameters assessed (three-way ANOVA; P>0.05; Table 1). The exception was a significant variety effect for the number of nymphs produced during a time span equaling the nymphal developmental time (FD) (F_2,147_ = 3.50; P = 0.033) which appears to be due to a discrepancy between the varieties MECH 12 and MECH 184. A significant experimental effect was calculated for the parameters FD, daily fecundity (DF), and total longevity (TL); however, no interaction among the different factors occurred.

**Table 1:** Performance of *Aphis gossypii* on *Bt* and non-*Bt* cotton varieties (n = 24 to 30).

Parameter a	Non-*Bt*			*Bt*			Variety effect e	Trans-formation effect e
	MECH 12	MECH 162	MECH 184	MECH 12	MECH 162	MECH 184		
**rm** b	0.333 (0.300 to 0.343)	0.366 (0.327 to 0.377)	0.371 (0.310 to 0.392)	0.335 (0.320 to 0.348)	0.355 (0.306 to 0.364)	0.368 (0.320 to 0.383)		
**FD** c	14.6 (13.1 to 16.1)	13.1 (11.5 to 14.6)	11.6 (9.9 to 13.4)	14.0 (12.0 to 16.0)	14.1 (12.2 to 16.0)	12.6 (10.9 to 14.4)	F_2,147_ = 3.503; P = 0.033	F_1,147_ = 0.460; P = 0.499
**DF** c	2.03 (1.82 to 2.23)	1.99 (1.77 to 2.20)	1.72 (1.54 to 1.89)	1.91 (1.67 to 2.14)	1.92 (1.69 to 2.15)	1.88 (1.66 to 2.10)	F_2,152_ = 1.454; P = 0.181	F_1,152_ = 0.001; P = 0.968
**TF** c	31.5 (26.9 to 36.1)	28.2 (23.6 to 32.8)	27.7 (22.8 to 32.7)	32.5 (26.5 to 38.5)	26.9 (22.1 to 31.6)	31.4 (26.2 to 36.6)	F_2,152_ = 1.985; P = 0.164	F_1,152_ = 0.496; P = 0.527
**D** d	6.3 (6.0 to 6.5)	6.0 (5.0 to 6.5)	6.5 (5.5 to 7.0)	6.0 (5.5 to 7.0)	6.0 (5.5 to 7.0)	6.0 (5.0 to 6.5)	W = 0.020	W = 0.055
							P = 0.889	P = 0.815
**AL** d	18.3 (14.5 to 21.0)	15.0 (13.5 to 18.5)	18.0 (15.0 to 21.5)	17.5 (14.5 to 23.0)	15.8 (13.0 to 18.0)	19.8 (15.3 to 23.3)	W = 1.155	W = 0.157
							P = 0.385	P = 0.759
**TL** d	24.0 (21.0 to 27.0)	21.0 (18.5 to 24.5)	24.0 (17.5 to 27.0)	22.8 (19.0 to 29.0)	21.5 (19.0 to 24.5)	25.3 (19.5 to 28.5)	W = 1.492	W = 0.375
							P = 0.255	P = 0.571

Bioassays were performed at 25°C±1°C day/20°C±1°C night, 70%±10% r.h. and a 16-h photoperiod.

Printed estimate refers to median and variability to first to third quartile in case of Cox-proportional hazard analysis, and to 95% confidence interval of the mean otherwise.

a (r_m_) intrinsic rate of increase (days); (FD) number of nymphs produced during D; (DF) daily fecundity; (TF) total fecundity; (D) nymphal developmental time (days); (AL) adult longevity (days); (TL) total longevity (days).

b bootstrap percentile method.

c 3-way ANOVA with experiment, cotton variety and *Bt*-transformation as factors.

d Cox-proportional hazard analysis with experiment, cotton variety and *Bt*-transformation as factors.

e F = Test statistic for F-distribution; W = Wald test statistics.

### Trichome density

**Figure 1:**
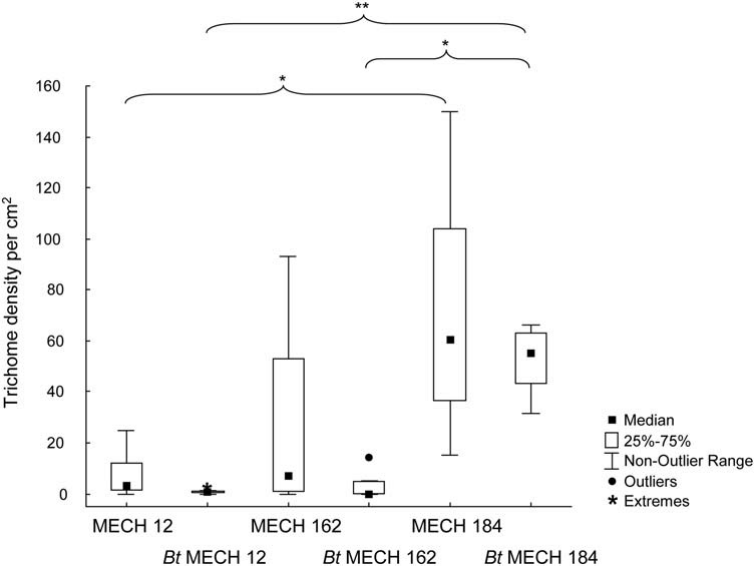
Trichome density on the lower surface of *Bt* and non-*Bt* cotton plants. Boxplot figures showing the median trichome density per cm^2^ (n = 6 to 8). Brackets indicate a significant difference between two treatments; *, P<0.05; **, P<0.01. The outlier range is the range of values that fall above the upper outlier limit (+1.5×the height of the box) and below the lower outlier limit (−1.5×the height of the box).

There was a significant difference in the trichome density among the six different cotton plants (Kruskal-Wallis ANOVA; χ^2^ = 23.07; df = 5; P = 0.033) (Fig. 1). Conducting pair-wise comparisons, a significantly greater trichome density was found for *Bt* MECH 184 compared to the other two *Bt* cotton varieties (Mann-Whitney U-test; *Bt* MECH 12; U = 0.00; P = 0.015 and *Bt* MECH 162; U = 0.00; P = 0.010). For the control varieties a higher trichome density was observed for MECH 184 compared to MECH 12 (U = 2.00; P = 0.010). No difference in the trichome density was observed between *Bt* and non-*Bt* leaves for any of the three pairs (P>0.05). Remarkably, *Bt* cotton plants showed a considerably reduced variability in trichome densities compared to *Bt* plants (Fig. 1).

### Quantification of Cry1Ac protein in leaves and aphids

Leaves of cotton plants on which aphids had fed during the bioassay in which aphid performance was evaluated, expressed the Cry1Ac protein at the following levels (mean±SE); *Bt* MECH 12: 0.58±0.060 µg Cry1Ac/g f.w., *Bt* MECH 162: 0.73±0.089 µg Cry1Ac/g f.w. and *Bt* MECH 184: 0.82±0.065 µg Cry1Ac/g f.w.

All ELISA readings revealed that aphids that had been kept on *Bt* cotton did not contain detectable Cry1Ac protein; i.e. readings were below the limit of detection (LOD) of 0.002 µg/g f.w. Leaves from which aphids were collected for ELISA analysis expressed the following amounts of Cry1Ac: *Bt* MECH 12: 0.34±0.100 µg Cry1Ac/g f.w., *Bt* MECH 162: 0.62±0.191 µg Cry1Ac/g f.w. and *Bt* MECH 184: 0.38±0.156 µg Cry1Ac/g f.w. As expected, none of the non-*Bt* cotton leaves or aphid samples contained any *Bt* protein.

### Sugar analyses in honeydew

As it was not possible to determine the exact amount of sugar in honeydew samples in µg/ml, the sugar composition was presented as percentage of total sugar content.

**Figure 2:**
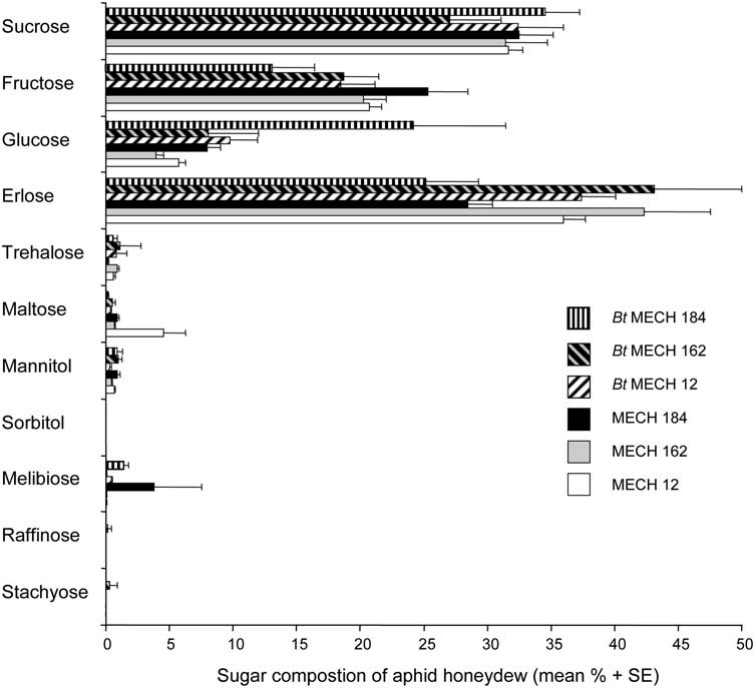
Relative sugar composition (mean percentage+SE) of *Aphis gossypii* honeydew. Honeydew was collected over a 5 to 6 h interval from either *Bt* (*Bt* MECH 12, *Bt* MECH 162, *Bt* MECH 184) or the corresponding non-*Bt* cotton plants (MECH 12, MECH 162, MECH 184) (n = 5 to 7). Lactose, melezitose, mannose, rhamnose, and galactose were not found in any of the samples.

A total of eleven sugars were found in the aphid honeydew. Dominant were the phloem sugars, sucrose and fructose, as well as the aphid-synthesized sugar erlose, that together made up 73% (*Bt* MECH 184) to 94% (MECH 162) of the total sugar content. Smaller amounts of glucose, trehalose, maltose, mannitol, melibiose, and stachyose were detected in all samples. Sugar composition appeared to differ between *Bt* and non-*Bt* plants as well as among varieties (Fig. 2). Whereas, fructose levels were higher in the honeydew from non-*Bt* cotton, glucose was amplified in honeydew from *Bt* cotton (especially for *Bt* MECH 184). Interestingly, erlose levels differed noticeably among the cotton varieties. While it was the dominant sugar in the honeydew collected from MECH 162 and *Bt* MECH 162, a much lower proportion of erlose was found in the honeydew of MECH 184 and *Bt* MECH 184. The greatest proportion of maltose was detected in MECH 12 and sorbitol was measured in all non-*Bt* samples (even though at very low levels) but in none of the *Bt* varieties. Raffinose occurred in just two out of the six treatments (MECH 12 and *Bt* MECH 162).

**Figure 3:**
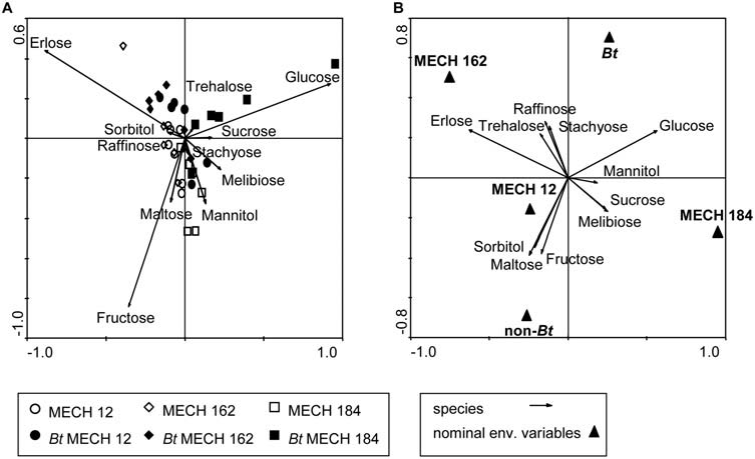
Distribution of sugar composition of honeydew samples from *Aphis gossypii*. Aphids were fed on *Bt* (*Bt* MECH 12, *Bt* MECH 162, *Bt* MECH 184) or the corresponding non-*Bt* cotton plants (MECH 12, MECH 162, MECH 184). Distribution of sugar composition shown in (A) the ordination biplot of a Principal Component Analysis (PCA) (eigenvalues: axis 1: 0.560, axis 2: 0.193) and (B) a Redundancy Analysis (RDA) (eigenvalues: axis 1: 0.249, axis 2: 0.066). The straight lines of the vectors represent the influence of the different sugars (species; Fig. 3 A; B), the triangles the centroids of the environmental variables (variety and *Bt*; Fig. 3 B). Sugars were expressed as percentage of total sugar.

Performing a Principle Components Analysis (PCA) to visualize the data, a negative correlation between the sugars with the greatest influence on data variability (those with the longest vector, namely erlose, glucose, and fructose) could be observed. Glucose, sucrose, and trehalose were positively correlated with each other and negatively with fructose, maltose, and raffinose (Fig. 3 A). A positive correlation among the amount of erlose and sorbitol and a negative correlation with melebiose, stachyose, and mannitol were observed in the data set. Looking at clusters in the biplot graphic, it was assumed that the first axis was best explained by the factor cotton variety (56%) and the second axis by the factor *Bt*-transformation (19%; Fig. 3 A). Performing a Redundancy Analysis (RDA) to analyze the influence of the evaluated explanatory variables [transformation (*Bt*/non-*Bt*) and variety (MECH 12, MECH 162, MECH 184)] showed a strong positive correlation between erlose, trehalose, raffinose, and stachyose with the variety MECH 162, and a negative correlation with the variety MECH 184 (Fig. 3 B). Glucose, trehalose, raffinose, and stachyose were positively correlated with *Bt* cotton plants, and negatively with the variety MECH 12. The sugars mannitol, melebiose, and sucrose were positively correlated with the variety MECH 184 and negatively with the variety MECH 162. Sorbitol, maltose and fructose were positively correlated with the variety MECH 12 and non-*Bt* cotton plants and negatively with *Bt* plants. The Monte Carlo permutation test revealed a significant difference in sugar composition of honeydew due to the variety MECH 184 (F = 8.60; P = 0.001) as well as the *Bt* transformation (F = 3.97; P = 0.004; Fig. 3 B). The fact that the first two axes of the RDA only explained 32% of the variance (first axis = 0.249; second axis = 0.066), indicated that there were variables other than transformation and variety that could explain the differences in honeydew sugar composition.

## Discussion

Aphid performance did not differ between *Bt* and non-*Bt* cotton plants and none of the aphid life-table parameters assessed was influenced by the expression of the *Bt* protein. However, there was a significant difference among cotton varieties for the number of nymphs produced during a time span equaling the nymphal developmental time (FD). Furthermore, a slight variation in the r_m_-values suggested a small difference among cotton varieties. As the r_m_ is difficult to interpret, the formula 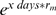 was used to calculate aphid population growth during one week. According to this formula, a population would have increased by a factor of ten on *Bt* and non-*Bt* cotton plants of the variety MECH 12 while population increase would have been more pronounced on the varieties MECH 162 (factor 12 or 13) and MECH 184 (factor 13). To detect differences among cotton varieties is not surprising since disparities are known to be caused by different plant characteristics, e.g. gossypol level [31] and trichome density [32]. The latter effect was also found in our study. While a previous study reported differences in the trichome density between *Bt* and non-*Bt* cotton plants [33], this could not be confirmed for the three cotton varieties used in our study. It was noticeable, though, that the trichome density of *Bt* plants showed considerably less variation as compared to non-*Bt* plants. The reasons for this discrepancy are not known.

While a previous greenhouse study addressing the performance of *A. gossypii* on *Bt* and non-*Bt* cotton plants showed a variation in some life-table parameters among three consecutive generations [34], studies comparing aphid populations in *Bt* and non-*Bt* cotton fields gave inconsistent results. While some studies recorded no difference in aphid populations [35]–[37], others found either increased [38], [39] or decreased aphid densities [40] in the *Bt* crop. Given the results from the greenhouse/climate chamber studies, changes in aphid populations in cotton fields are unlikely directly attributable to the expression of the *Bt* Cry protein. Rather they may be due to other confounding factors, such as changes in the use of insecticides or increased overall health of the *Bt* crop [4], [41]. In *Bt* maize, a study has shown aphids to perform significantly better (expressed as increase in aphid numbers) on different *Bt* maize varieties with different transformation events than on their respective non-transformed control varieties [42]. While slight *Bt* maize effects on aphid performance had earlier been reported [43], other greenhouse studies did not show evidence of such effects [15], [44], [45]. In the field, no or only minimal differences in aphid densities on *Bt* and non-*Bt* maize have been recorded [46], [47].

Our ELISA analyses revealed that none of the aphid samples contained detectable *Bt* protein. This finding is in accordance with many other studies which reported either no *Bt* protein or only trace amounts of protein in sap-sucking insects of the order Hemiptera after feeding on different *Bt* plants, including maize [15]–[17], [19], oilseed rape [18], cotton [20], and rice [48], [49]. In contrast to the studies listed above, Burgio et al. [22] detected 3% of the Cry1Ac protein content expressed by *Bt* oilseed rape (plant expression level: 64 ng Cry1Ac/g f.w.) in all of four aphid samples that had been collected in the greenhouse, but only in one out of eight samples collected from plants kept in a climate chamber (2% of the Cry1Ac protein content expressed by *Bt* oilseed rape). Even higher levels were reported from greenhouse studies by Zhang et al. [21] conducting ELISA analyses of aphids which previously had fed on a medium (plant expression level: 49 ng Cry1A/g f.w.) or a high (plant expression level: 94 ng Cry1A/g f.w.) expressing *Bt* cotton line. Surprising in this study was the fact that all ten aphid samples contained *Bt* protein after feeding on the medium *Bt*-expressing line, compared to only four out of ten samples after feeding on the high *Bt*-expressing line. Furthermore the positive aphid samples from the medium expressing *Bt* cotton plants contained 12% of the *Bt* content present in the plant while the aphid samples collected from the high-expressing *Bt* cotton contained only 4% of the *Bt* amount found in the plants. It is notable that (with one exception) all positive samples in the two studies listed above were collected in the greenhouse. We argue that one likely reason for the *Bt* proteins detected in these studies is contamination of the samples by other herbivores such as spider mites or thrips or their feces which contain large amounts of *Bt* protein [19], [50], [51]. In a preliminary study, we had collected aphids from *Bt* cotton that was contaminated with thrips. Great care was taken to check all aphid samples under the binocular microscope, both before releasing the aphids on the cotton plants and again on recollection from the plants to ensure that all herbivores other than aphids were removed. Nevertheless subsequent ELISA analyses still detected some *Bt* protein in eleven out of twelve samples (0.02 to 0.06 µg Cry1Ac/g f.w. aphids, corresponding to 13–25% of the amount detected in cotton leaves; Lawo unpublished data). These protein levels were 10 to 30 times higher than the limit of detection of the ELISA used here. These findings underline that very low levels of contamination are sufficient to produce false positives, especially in samples that contain only traces, or no *Bt* protein, like aphids.

To prevent potential thrips or spider mite infestations of the plants and thus contamination of the aphid samples, our experiment was conducted in a climate chamber instead of a greenhouse. No *Bt* protein could be detected in any of the aphid samples collected in the climate chamber, even though the *Bt* cotton plants were expressing similar levels of *Bt* protein compared to greenhouse-grown plants. It can thus be concluded that aphids feeding on *Bt* cotton do not ingest measurable amounts of *Bt* protein and consequently cannot pass it on to their natural enemies. The Cry1Ac expression levels found in the climate chamber were lower than those reported for the same cotton varieties from an Indian field study [52]. Comparisons of *Bt*-expression levels remain difficult, however, since they vary with plant age and various environmental factors [53]. Moreover, ELISA results commonly differ between studies conducted with different kits, extraction methods and purified proteins used as standards. Due to the fact that the ELISA measurements were very sensitive (with an LOD of 0.002 µg/g f.w.), it can be concluded that aphids ingest no or negligible amounts of Cry1Ac when feeding on *Bt* cotton even if the expression levels in the field might be higher. Consequently, negligible amounts of Cry1Ac will be passed from aphids to higher trophic levels.

In the case of phloem feeders such as aphids, the performance of natural enemies can also be influenced indirectly through the nutritional value of the honeydew [54]. The suitability of honeydew as a sugar source can be affected by nutrient composition [54], as well as by the presence of secondary metabolites [23] or insecticidal proteins [55], [56]. Honeydew sugar composition has been found to vary not only among plant species and different honeydew-producing insects [29], [57]–[59], but also with the insect's developmental stage [60], [61] and age [62], ant attendance [63], [64], the presence of bacterial symbionts in the digestive tract [65] as well as the rate and duration of insect infestation on the plant [66].

Using multivariate statistics to analyze the sugar composition of honeydew collected from *A. gossypii* feeding on the different varieties of *Bt* and non-*Bt* cotton, both variety and transformation were found to have a significant influence. This bioassay is the first case in which variation in honeydew sugar composition can be attributed to the latter factors. The fact that honeydew sugar composition differs among cotton varieties is not surprising given the fact that the plants generally differ in a range of parameters [31], [32]. The variation in sugar composition due to transformation might be explained by differences between *Bt* cotton plants and their non-transformed counterparts that go beyond the intentionally introduced genes. The selection process taking place after transformation induces these additional differences in the plant material. The changes are unlikely caused by the expression of the *Bt* protein since it could clearly be shown that the aphids are not exposed to the insecticidal compound. The fact that more glucose was detected in honeydew collected from *Bt* plants (especially *Bt* MECH 184) compared to that from the controls may be based on variation in phloem sap composition. Alternatively, it may indicate that the carbohydrate assimilation by the aphids was somewhat affected [67]. Either way, it is interesting that this difference did not have any influence on aphid performance. However, for a proper evaluation of the nutritional quality of the phloem sap the amino acid composition should directly be examined. In contrast to our results, Faria et al. [42] did not find any difference in sugar composition in aphid honeydew collected from different *Bt* and non-*Bt* maize varieties. However, the authors reported marginal differences in the amino acid composition.

Since the plant-derived sugars, mainly sucrose, glucose and fructose, are the most suitable for honeydew-feeding natural enemies [27], honeydew from the cotton variety MECH 184 might have increased nutritional properties compared to the other two varieties tested. However, we believe that this effect should not be overestimated since cotton features multiple nectar and extra-floral nectar sources [68], which are frequently exploited by parasitoids [69]. When given a choice, parasitoids may ignore honeydew, and select extrafloral nectar. Among *Microplitis croceipes* (Cresson) (Hymenoptera: Braconidae) collected from fields with and without nectar sources, only individuals from fields lacking nectar contained honeydew-specific sugars (Williams and Wäckers unpublished data).

Our studies allow the conclusion that aphid performance is not affected by Cry1Ac-expressing *Bt* cotton plants. This, together with the fact that aphids do not ingest the insecticidal protein when feeding on *Bt* cotton, indicates that aphid antagonists are unlikely to be affected either directly or indirectly when attacking aphids in a *Bt* cotton field and that the biological control function they provide should not be compromised. This is also true for generalist predators that attack other herbivores on cotton, given the Lepidoptera-specificity of Cry1Ac [12], [13]. In accordance to our laboratory studies, Bambawale et al. [35] observed no difference in cotton aphid abundance between Indian fields with *Bt* cotton (MECH 162) and its corresponding near isoline under integrated pest management. Consequently, aphids are likely to remain under biological control in *Bt* cotton, which is important for the sustainable deployment of this technology.

## Materials and Methods

### Aphis gossypii

*Aphis gossypii* were provided by Syngenta (Stein, Switzerland) and were subsequently reared continuously on cotton (*Gossypium hirsutum*) in a climate chamber at 25±1°C, 70±10% r.h., and a 16-h photoperiod.

### Cotton plants

Three *Bt*-transgenic *Gossypium hirsutum* varieties (MECH 12, MECH 162, and MECH 184; event BG-I, Mahyco Seeds Ltd.), expressing the Cry1Ac protein (*Bt* plants) and their corresponding non-transformed near isolines (non-*Bt* plants) were used in the bioassays. All three *Bt* varieties have been commercially grown in India since 2002.

Plants were grown individually in humus-rich sterilized soil in plastic pots (3 litre) and fertilized weekly with 10N∶10P∶8K at a concentration of 20 ml/l. Plants were grown in a climate chamber, illuminated by metal halide lamps (EYE Clean-Ace lamps, MT400/DL/BH; Iwasaki Electric Co., Ltd.) at 25°C±1°C day/20°C±1°C night, 70%±10% r.h. and a 16-h photoperiod (20,000 lux during daytime). Changes between day and night conditions occurred gradually to simulate natural dusk and dawn. Metal halide lamps featured a light color close to daylight. To guarantee stable humidity, plants were placed individually in plant dishes (16 cm in diameter; 3 cm high). Plants were watered once a day until the eight-leaf stage, after which they were watered twice daily. Prior to bioassays cotton plants were controlled visually for any insect damage to avoid unintended induction of the cotton innate resistance mechanisms.

### Experimental set-up

For one bioassay, 15 metal trays (45×90 cm) were placed in two rows on the floor of the climate chamber, each containing six, three-week old plants (two to five true leaves). The plants, one of each *Bt* and non-*Bt* variety, were ordered randomly per tray (complete block design). The bioassay was repeated twice resulting in a total of 30 plants per treatment. Environmental conditions were the same as described above for cultivation of the plants.

### Aphid performance

A group of approximately 50 reproductive aphids from the permanent culture were allowed to settle on a plant and to give birth to nymphs. After 6 h, two to three newborn nymphs (F_1_) were brushed carefully onto the last fully expanded leaf of the respective variety and covered with a clip cage (2 cm in diameter; 1 cm high). Clip cages featured a hole sealed with fine-mesh netting to provide air-circulation. After three days, surplus nymphs were removed at random, to ensure that the performance of a single nymph was monitored on each plant. Every morning and evening, aphid mortality was recorded. After reaching adulthood the F_1_ nymphs were counted and removed daily. This procedure was conducted until the aphid died.

The following aphid life-table parameters were obtained this way: nymphal developmental time (time from birth to first nymphal production; D), number of nymphs produced during a time span equal to D starting at nymph production (FD), mean reproductive rate during the reproductive period observed (daily fecundity; DF), total fecundity per female during the period observed (TF), adult longevity (AL), total longevity (TL), and intrinsic rate of population increase (r_m_). The r_m_ was estimated based on the daily age-specific fecundity (m_x_) and the age-specific survival rate (l_x_) and using the equation of Birch [70] (1):
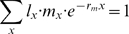
(1)Aphids that died before producing any nymphs were excluded from the analysis. Aphids which were lost during the observation period were censored.

### Trichome density

After the death of the aphid, the leaf below the one exposed to the aphid was collected and stored at −20°C until further examination. For each leaf, three trichome counts were taken from a central section of the lower surface, excluding the primary and secondary leaf veins. Depending on trichome density, measurements were done on an area of 16 or 64 mm^2^ (for high or low trichome density, respectively) under a binocular microscope (WILD, Heerbrugg) with the help of an ocular measuring grid (Leica). Six to eight samples were counted per treatment and subsequently data were calculated as trichome density per cm^2^.

### Quantification of Cry1Ac protein in leaves and aphids

To confirm Cry1Ac expression of the transgenic cotton plants used in both bioassays, a total of 12 to 13 leaves per variety of the *Bt* plants and three leaves per variety of the non-*Bt* plants were collected after the end of the bioassays. Approximately 100 mg fresh weight (f.w.) of the leaves on which *A. gossypii* had fed were sampled, flash frozen, weighed, lyophilized and weighed again (approximately 20 mg dry weight).

To quantify the level of Cry1Ac in *A. gossypii*, 60 to 70 reproductive aphids were brushed on the last fully expanded leaf of three-week old *Bt* or non-*Bt* cotton plants and allowed to reproduce for five weeks. Subsequently, leaves infested with aphids were transferred to two to three-week old cotton plants of the same variety and transformation status and reared under the same climatic conditions. Aphids were allowed to settle and reproduce for one to two additional weeks. Thereafter, 30 to 80 mg of aphids was collected using a flexible tube on which gauze and a tip were attached. Before weighing and lyophilizing, each sample was checked under a binocular microscope to confirm that there was no contamination with other pests (e.g., spider mites or thrips) or leaf pieces. Three to five samples per *Bt* variety and one per non-*Bt* variety were analyzed. From the same plants on which aphids were kept, samples from two of the most heavily infested leaves were also taken for *Bt* protein measurements.

The amount of Cry1Ac protein in leaf and aphid material was measured using an enzyme linked immuno-sorbent assay (ELISA) from Agdia (Elkhart Indiana, USA). After adding phosphate buffered saline with Tween buffer (PBST, provided in the kit) at a ratio of 1∶10 (sample material f.w.∶buffer) and a 5 mm tungsten carbide bead, leaf samples were macerated for 100 sec at 15 Hz and aphid samples for 40 sec at 30 Hz, using a mixer mill MM300 (Retsch, Haan, Germany) fitted with 24 tube-adapters (Qiagen, Hombrechtikon, Switzerland). Samples were centrifuged for 5 min at 13,000×g and leaf samples were diluted 1∶15 with PBST, while aphid samples were not diluted. Subsequently, instructions from the kit were followed. After stopping the color development with 3 M sulfuric acid, spectrophotometric measurements were conducted with a microtiter plate reader (SpectrafluorPlus, Tecan, Männedorf, Switzerland) at 450 nm. A standard curve with purified high quality Cry1Ac provided by M. Pusztai-Carey (Dept. Biochemistry, Case Western Reserve University, Cleveland, OH, USA) was established. Concentrations were calculated using linear regression analysis. The limit of detection for aphid extracts was calculated by multiplying three times the standard deviation of eleven buffer-only and control ODs with the slope of the standard curve. Based on µg/g f.w., the limit of detection (LOD) was 0.002.

### Sugar analysis of aphid honeydew

For sugar analysis, approximately 50 mg of aphids was collected from the rearing colony in a clip cage (5.2 cm in diameter; 1 cm high) in the afternoon and allowed to settle overnight on the youngest fully expanded cotton leaf. The cage was removed the following morning and the aphids were allowed to settle one additional day.

For honeydew collection, Petri dishes were placed under the aphid infested cotton leaves for 5 to 6 h. Thereafter, honeydew-sprinkled Petri dishes were placed upside down at 23±1°C and 85±5% r.h., with a water-saturated piece of cotton wool on the bottom. After 2 h, when the viscosity of the honeydew was reduced through hygroscopy, approximately 0.5 µl honeydew was collected with 5 µl micropipettes (Blaubrand, intra Mark). Subsequently, the honeydew was dissolved in 20 µl ethanol (70%) and stored at −80°C until further analysis.

For sugar analysis of aphid honeydew, five to seven samples per treatment were tested for 16 sugars: sucrose, fructose, glucose, erlose, trehalose, mannitol, sorbitol, melibiose, raffinose, stachyose, lactose, melezitose, mannose, rhamnose, maltose and galactose. Before analysis, samples were diluted 400-fold with 18 MΩ water and homogenized using a pestle. Subsequently, samples were filtered through a chromacol syringe filter. Samples were analyzed using a Dionex ICS 3000 Ion Chromatography system (Dionex Corp., Sunnyvale, CA, USA) and concentrations of the individual sugars were calculated using the program PEAKNET Software Release 5.1 as described by Steppuhn and Wäckers [71]. The limit of quantification for any honeydew sample was set at 0.001 µg. Measurements below 0.001 µg were set to 0.

### Data analysis

Since all assumptions were met, FD, DF, and TF were tested with a multivariate ANOVA including the factors *Bt*/non-*Bt*, variety, and experiment. Means were subsequently compared using the Tukey-Kramer Test. D, AL, and TL were analyzed by means of a Cox regression model including the factors *Bt*/non-*Bt*, variety, and experiment. Insects which were lost over the observation period were censored. To estimate the confidence intervals for the r_m_, the bootstrap percentile method with 10,000 resamples was performed [72].

Trichome density was compared among all treatments with a Kruskal-Wallis ANOVA followed by pair-wise comparisons using the Mann-Whitney U-test, adjusted for ties. The two-sided exact P-value was subsequently corrected with the Bonferroni-Holm procedure. Statistical analyses were conducted using the software package Statistica (version 6, StatSoft Inc., Tulsa, OK, USA) or R (bootstrap). The α-level was 5% in all statistical analyses.

To calculate the sugar composition of the different *Bt* and non-*Bt* varieties, firstly, the lengths of gradient was calculated. Since the value was <3, the use of linear models was justified. Therefore, an unconstrained linear ordination (indirect gradient analysis) using Principal Components Analysis (PCA) on sugar percentages (species data) was conducted to visualize the important patterns in the data. Additionally, the distribution of the sugar concentrations was investigated by constrained linear ordination (direct gradient analysis), using Redundancy Analysis (RDA) to analyze the variability between *Bt* and non-*Bt* plants and/or among varieties based on the composition of different sugars. The transformation (*Bt*/non-*Bt*) and variety (MECH 12, MECH 162, MECH 184) were used as explanatory variables. The significance of each axis of the RDA was tested using a Monte Carlo permutation test with unrestricted permutations (n = 999), followed by forward selection to determine the relative importance and significance of each environmental variable.

The software package CANOCO 4.5 was used to conduct the multivariate analysis [73].
